# Development of a multivariable model to predict vulnerability in older American patients hospitalised with cardiovascular disease

**DOI:** 10.1136/bmjopen-2015-008122

**Published:** 2015-08-27

**Authors:** Susan P Bell, John Schnelle, Samuel K Nwosu, Jonathan Schildcrout, Kathryn Goggins, Courtney Cawthon, Amanda S Mixon, Eduard E Vasilevskis, Sunil Kripalani

**Affiliations:** 1 Division of Cardiovascular Medicine, Department of Medicine, Vanderbilt University, Nashville, Tennessee, USA; 2 Division of General Internal Medicine and Public Health, Department of Medicine, Center for Quality Aging, Vanderbilt University, Nashville, Tennessee, USA; 3 Department of Biostatistics, Vanderbilt University Medical Center, Nashville, Tennessee, USA; 4 Center for Clinical Quality and Implementation Research, Nashville, Tennessee, USA; 5 Center for Health Services Research, Vanderbilt University, Nashville, Tennessee, USA; 6 Department of Veterans Affairs, Tennessee Valley Healthcare System—Geriatric Research Education and Clinical Center (GRECC), Nashville, Tennessee, USA; 7 Division of General Internal Medicine and Public Health, Department of Medicine, Section of Hospital Medicine, Vanderbilt University, Nashville, Tennessee, USA

**Keywords:** GERIATRIC MEDICINE

## Abstract

**Objectives:**

To identify vulnerable cardiovascular patients in the hospital using a self-reported function-based screening tool.

**Participants:**

Prospective observational cohort study of 445 individuals aged ≥65 years admitted to a university medical centre hospital within the USA with acute coronary syndrome and/or decompensated heart failure.

**Methods:**

Participants completed an inperson interview during hospitalisation, which included vulnerable functional status using the Vulnerable Elders Survey (VES-13), sociodemographic, healthcare utilisation practices and clinical patient-specific measures. A multivariable proportional odds logistic regression model examined associations between VES-13 and prior healthcare utilisation, as well as other coincident medical and psychosocial risk factors for poor outcomes in cardiovascular disease.

**Results:**

Vulnerability was highly prevalent (54%) and associated with a higher number of clinic visits, emergency room visits and hospitalisations (all p<0.001). A multivariable analysis demonstrating a 1-point increase in VES-13 (vulnerability) was independently associated with being female (OR 1.55, p=0.030), diagnosis of heart failure (OR 3.11, p<0.001), prior hospitalisations (OR 1.30, p<0.001), low social support (OR 1.42, p=0.007) and depression (p<0.001). A lower VES-13 score (lower vulnerability) was associated with increased health literacy (OR 0.70, p=0.002).

**Conclusions:**

Vulnerability to functional decline is highly prevalent in hospitalised older cardiovascular patients and was associated with patient risk factors for adverse outcomes and an increased use of healthcare services.

**Strengths and limitations of this study**

- Large prospective observational study that utilises the Vulnerable Elders Survey (VES-13) to assess the prevalence of vulnerability to functional decline in older adults admitted to the hospital with an acute cardiovascular event.
- In-depth sociodemographic measurements to examine associations with physical vulnerability that include health literacy, numeracy, social support, education, living and marital situation.
- Study examines relationship between vulnerability and depressive symptoms, cognition and frailty indices.
- The VES-13 does integrate self-perceived health, physical function limitations and IADL/ADL disabilities but does not include an objective measure, and due to the self-reported nature, the study excludes individuals who cannot communicate due to severity of the illness.
- This research was supported by National Institutes of Health/National Heart, Lung, and Blood Institute (NIH/NHLBI; R01 HL109388) and in part by the National Center for Advancing Translational Sciences (2 UL1 TR000445-06). SPB is supported by K12HD043483-11 from NIH/National Institute of Child Health and Human Development (NICHD) and by the Eisenstein Women's Heart Fund. AMS is a VA Health Services Research and Development Service (HSR&D) Career Development awardee at the Nashville VA. EEV is supported by NIH/National Institute on Aging (NIA) under Award Number K23AG040157 and the Veterans Affairs Clinical Research Center of Excellence, and the Geriatric Research, Education and Clinical Center (GRECC). The content is solely the responsibility of the author(s) and does not necessarily represent official views of the Department of Veterans Affairs and the NIH.

## Introduction

Cardiovascular disease (CVD) affects approximately 40 million individuals in the USA over the age of 65 years and is the leading cause of mortality.^1^ Hospitalisation for an acute cardiovascular event is a significant stressor and can lead to functional decline, both during the admission and at 12 months follow-up.^2^ Older adults who experience a decline in functional status are vulnerable to adverse health outcomes, including an increased risk of hospitalisation, institutionalization and mortality.^3–5^ The extent of vulnerable functional status in hospitalised cardiovascular (CV) patients, however, is poorly characterised. Currently, there is no widespread standard for assessment of vulnerable functional status in the hospital setting. Although clinicians are able to recognise severe geriatric impairments, their sensitivity to detect moderate impairments or change in functional impairment is imperfect.^6^ Unrecognised impairments may result in new functional needs that are unmet after discharge and an increased risk of rehospitalisation.^7^

Numerous multidimensional assessment tools have been developed to measure physical frailty or vulnerable functional status.^5^ ^8–15^ The majority have been developed for use in the ambulatory setting and range from a composite score of reported clinical deficits to physical performance-based criteria.^5^ ^11^ ^15^ An optimal assessment in hospitalised older adults would include an objective physical performance test^16^ ^17^ (eg, gait speed and hand grip strength). However, in the inpatient setting, these tests may not be feasible to administer across all patients due to constraints of bed rest, monitoring devices, continuous intravenous therapies, acute pain or discomfort, disability or other physical limitations, particularly among patients with CVD who may have recently undergone an invasive procedure such as radial or femoral access coronary angiography.^18–20^

One method developed and validated in community populations to identify older adults at risk of decline is a self-report instrument, the Vulnerable Elders Survey (VES-13).^21^ ^22^ The VES-13 is not affected by these restrictions and has been applied in the hospital setting to predict adverse events in older adults following traumatic injury.^23^ Using the VES-13, we sought to (1) determine the prevalence of vulnerability among older CV patients in the hospital setting, (2) to develop a multivariable prediction model to determine the association between VES-13 and prior healthcare utilisation, and the presence of concomitant patient risk factors that predict vulnerability and may adversely affect health outcomes.

## Methods

### Study design and participants

The Vanderbilt Inpatient Cohort Study (VICS) is a prospective longitudinal observational study that enrols patients admitted to medical or surgical units at the Vanderbilt University Hospital (VUH), Nashville, Tennessee, with a diagnosis of acute coronary syndrome (ACS) or acute decompensated heart failure (ADHF).^24^ Eligible participants presented initially to VUH or were transferred to VUH within 7 days of initial presentation to another hospital and met clinical criteria for intermediate to high likelihood of ACS or ADHF as defined by clinical signs and symptoms and indicators in the electronic medical record. All diagnoses were confirmed by a physician investigator.

Exclusion criteria were: unable to communicate in English, under hospice care, unstable psychiatric condition, inability to consent or participate due to medical condition or treatment (significant dementia, sedated), uncooperative or in police custody, visual or hearing impairment precluding participation, and already enrolled in VICS or a conflicting study.^24^ Patients who were delirious (acutely confused and screened positive by the Brief Confusion Assessment Method)^25^ or too ill (arterial balloon pump, receiving intravenous inotropes, intubated and/or sedated) to participate early during hospitalisation were reassessed for up to 7 days for potential eligibility. The sample for this current study included individuals 65 years and older enrolled consecutively between October 2011 and August 2013 from VICS, and who had completed the baseline assessment. All participants provided informed consent.

### Study procedures

A detailed description of study procedures has been published previously.^24^ After obtaining consent, research assistants completed an inperson interview with each participant and data were entered directly into the REDCap^26^ platform via a tablet computer. Baseline assessment includes demographics, education, cognition, psychological and social factors, prior healthcare utilisation and practices, and vulnerable functional health status using the VES-13.^22^ Individuals are asked to respond to 13 items that included age (scored 1 point for age 75–84, 3 points for 85+), self-rated health status (1 point), physical activity limitations (stooping, lifting, walking ¼ mile, grasping and heavy housework; 1 point each for difficulty or inability to do; maximum 2 points), and limitations in five representative activities of daily living (ADLs) and independent activities of daily living (IADLs), including shopping, managing money, walking across a room unaided, light housework and bathing (1 point each for inability to do or needing assistance, maximum 4 points). A completed survey resulted in a score of 0–10, with higher scores indicating more vulnerability.^22^ To compare the prevalence of vulnerability with frailty, the two self-reported components of the fried frailty phenotype were assessed. Exhaustion was measured using the Center for Epidemiologic Studies Depression Scale (CES-D).^5^ The shrinkage component of frailty was measured by self-reported unintentional weight loss >5% of body weight or ≥10 lbs over the preceding 6 months.^5^

To assess prior healthcare utilisation, all individuals were asked whether they had a regular physician and how many clinic visits, emergency room (ER) visits and hospital admissions they had done in the prior 12 months.

To understand whether vulnerability was associated with known markers of poor outcomes following hospitalisation, we also assessed cognition (Short Portable Mental Status Questionnaire (SPMSQ)),^27^ health literacy (short Test of Functional Health Literacy in Adults (s-TOFHLA)),^28^ numeracy (shortened 3-item version of the Subjective Numeracy Scale (SNS)),^29^ ^30^ depressive symptoms (Patient Health Questionnaire (PHQ-8)),^31^ perceived social support (ENRICHD Social Support Inventory (ESSI)),^32^ and number and frequency of contact with close friends and family members (items from the Health and Retirement Survey (HRS)^33^ and the Midlife Development in the United States (MIDUS) survey).^34^

## Statistical analysis

### Prevalence of frailty and vulnerability

To summarise the VES-13 distribution, scores of 0–2 were classified as non-vulnerable, scores of 3–6 were classified as having vulnerable functional health status, and scores of 7–10 were classified as extremely vulnerable to functional health status decline, with the latter two classes comprising the vulnerable class consistent with prior literature.^21^ ^22^

Within the vulnerability classes, continuous baseline variables are expressed as centiles (ie, 10th, 50th, 90th), and categorical variables as frequencies and percentages.

### Factors associated with frailty and vulnerability

To test unadjusted (bivariate) associations between vulnerability and each of the risk factors (demographics, education, cognition, psychosocial factors, and prior healthcare utilisation and practices), Pearson χ^2^ tests were performed for categorical variables, Kruskal-Wallis tests for continuous variables, and proportion trend tests were used for ordinal variables. All unadjusted analyses are susceptible to confounding and should be interpreted with caution.

Proportional odds multivariable regression analyses examined the independent associations of prespecified factors with the degree of vulnerability. These factors included age, sex, race, diagnosis, frailty, education, depression, health literacy and numeracy, cognition, social support, marital and living status, and prior healthcare utilisation. In the primary model, the outcome was VES-13 score as it generally appears in the literature, in which patients receive points for their age; this model did not include age as a covariate since an assumption of the model would be that all associations are related to age. In a secondary model (sensitivity analysis), we used VES-12 (which excludes age from calculation of the VES score) as the outcome^23^ and included age as a predictor.

By default, continuous predictors were modelled non-linearly with restricted cubic splines using three knots. Those variables for which there were little to no evidence of non-linearity, according to likelihood ratio tests were modelled linearly. Parameter estimates were exponentiated to obtain ORs for higher vulnerability scores along with their corresponding 95% CI. To avoid casewise deletion of records with missing covariates, we employed multiple imputation with five imputation data sets via predictive mean matching. Less than 1% of all variables were missing across the data set.^35^ All analyses were performed using the R V.2.15.1 (R Development Core Team, Vienna, Austria).

## Results

### Prevalence of vulnerability and frailty

The median VES-13 score was 3.0 (IQR 1.0, 6.0). Vulnerability was present in 54% of patients, with 30% of patients classified as moderately vulnerable (score 3–6) and an additional 24% classified as extremely vulnerable (score ≥7). Prevalence of exhaustion and shrinkage frailty criteria was also extremely high in this hospitalised population with 62.9% of individuals reporting one or both criteria. A total of 41.8% met criterion for exhaustion only, 6.7% met criterion for shrinkage only and 14.4% met criteria for both. Prevalence of exhaustion and/or shrinkage frailty criteria increased substantially as the level of vulnerability increased from non-vulnerable (48.8% frailty) to moderate (72% frailty) to extremely vulnerable (79.2% frailty).

### Patient characteristics and unadjusted analysis

Among 445 individuals aged 65 years and older included in this analysis, the median age was 71, 47% were women and 10% were African–Americans. Overall, 59% of the sample had a diagnosis of ACS, 32% had a diagnosis of ADHF and 9% were diagnosed with both ACS and ADHF.

We use unadjusted associations to describe marginal relationships between risk factors and the VES-13 (after coarsening); however, such results are susceptible to confounding and observed relationships should not be overinterpreted. We report adjusted analyses in the next subsection. In unadjusted analyses, increasing vulnerability was associated with female sex, fewer years of education, marital status, difficulty paying bills, reduced cognition and a diagnosis of ADHF, but was not associated with race, emotional or social support, or living alone (table 1). Notably, a difference in median age was seen between non-vulnerable and moderately vulnerable (3–6) individuals, but not between moderately vulnerable (3–6) and extremely vulnerable (7–10) individuals. Individuals classified as vulnerable did not report significantly different levels of social and family support and contact compared with those who were non-vulnerable. A significant association was observed between increasing VES-13 scores and cognitive impairment, lower numeracy, and lower health literacy. Extremely vulnerable adults (score 7–10) had a high (37%) prevalence of inadequate health literacy. Higher VES-13 scores were also associated with increasing prevalence of moderate and severe depressive symptoms (non-vulnerable 18.9%, moderately vulnerable 27.3% and extremely vulnerable 50.9%).

**Table 1:** Baseline characteristics by vulnerability

		VES-13 category			
	All	Non-vulnerable	Moderately vulnerable	Extremely vulnerable	p Value
Characteristic	N=445	N=207 (46%)	N=132 (30%)	N=106 (24%)	
Age	71 (66–82)	70 (66–78)	74 (66–84)	73 (67–84)	<0.001*
Diagnosis					<0.001†
ACS only	59.3%	76.3%	54.5%	32.1%	
Heart failure only	31.9%	17.4%	34.1%	57.5%	
Heart failure and ACS	8.8%	6.3%	11.4%	10.4%	
Sex, female	47.4%	39.6%	47.0%	63.2%	<0.001†
Race					0.396†
Caucasian	87.4%	90.3%	86.3%	83.0%	
African–American	9.9%	7.2%	10.7%	14.2%	
Other	2.7%	2.4%	3.1%	2.8%	
Education, years	13 (10–18)	14 (11–18)	14 (11–18)	12 (8–18)	0.006*
Marital status					0.031†
Married/living with partner	62.5%	66.7%	62.9%	53.8%	
Unmarried	13.7%	15.0%	13.6%	11.3%	
Widowed	23.8%	18.4%	23.5%	34.9%	
Living alone	24.5%	22.2%	25.0%	28.3%	0.490†
Frailty indices					
Exhaustion only	41.8%	32.4%	47.7%	52.8%	<0.001†
Weight loss only	6.7%	7.2%	7.6%	4.7%	0.631†
Exhaustion and weight loss	14.4%	9.2%	16.7%	21.7%	0.008†
Social support					
MIDUS sum score	15 (9–22)	14 (9–21)	15 (10–22)	16 (8–22)	0.498*
HRS sum score	9 (4–25)	9 (4–24)	9 (3–27)	9 (4–27)	0.910*
ESSI sum score	27 (20–30)	28 (20–30)	27 (21–30)	28 (21–30)	0.757*
Paying bills					0.005†
Somewhat/very difficult	37.1%	31.4%	34.0%	51.7%	
No difficulty/minimal difficulty	61.8%	67.6%	65.2%	46.1%	
Not sure/refused	1.1%	1.0%	0.8%	2.2%	
s-TOFHLA category					<0.001†
Inadequate	19.7%	13.1%	16.3%	36.6%	
Marginal	11.4%	10.6%	14.6%	8.9%	
Adequate	69.0%	76.3%	69.1%	54.5%	
Subjective Numeracy Score	5 (3–6)	5 (3–6)	5 (3–6)	4 (2–6)	<0.001*
Cognitive impairment	11.7%	7.2%	14.4%	17.0%	0.021†
PHQ-8 Depression Score	6 (2–15)	4 (1–12)	7 (3–14)	10 (4–18)	<0.001*

Continuous variables: median (10th–90th centiles); VES-13 categories: non-vulnerable (score 0–2), moderately vulnerable (score 3–6), extremely vulnerable (score 7–10).

*Kruskal-Wallis test.

†Pearson test.

ACS, acute coronary syndrome; ESSI, ENRICHD Social Support Inventory; HRS, Health and Retirement Survey; MIDUS, Midlife Development in the United States; PHQ, Patient Health Questionnaire; s-TOFHLA, short form of the Test of Functional Health Literacy in Adults; VES, Vulnerable Elders Survey.

Assessment of prior healthcare utilisation (table 2) demonstrated that there was no significant difference in the prevalence of having a regular physician. Increasing vulnerability was, however, associated with increased use of healthcare services, including a greater number of clinic visits, ER visits and hospitalisations in the 12 months preceding the index hospitalisation.

**Table 2:** Healthcare utilisation by vulnerability

		VES-13 category			
	All	Non-vulnerable	Moderately vulnerable	Extremely vulnerable	p Value
	N=445	N=207	N=132	N=106	
Regular physician	97.1%	95.2%	98.5%	99.1%	0.080*
Clinic visits in prior 12 months	7 (2–20)	6 (2–15)	8 (3–24)	10 (4–24)	<0.001†
ER visits in prior 12 months	1 (0–4)	1 (0–3)	1 (0–4)	2 (0–5)	<0.001†
Hospitalisations in prior 12 months	1 (0–4)	0 (0–2)	1 (0–4)	2 (0–5)	<0.001†

Continuous variable centiles: 10th, 50th, 90th.

*Pearson test.

†Kruskal-Wallis test.

VES, Vulnerable Elders Survey.

### Multivariable models

**Figure 1:**
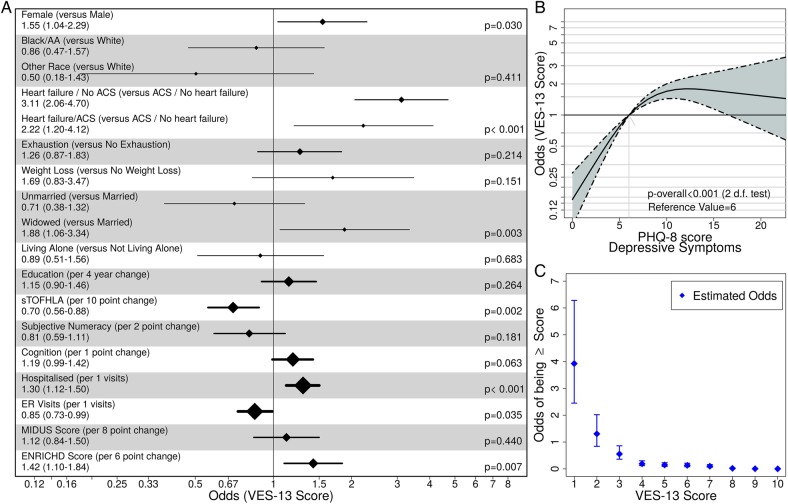
(A) Primary Multivariable Proportional Odds Model for VES-13 score demonstrating linear associations per unit increase of VES-13 score; (B) non-linear relationship of increasing depressive symptoms (PHQ-8) score with odds of increased VES-13 score; (C) estimated intercepts from the proportional odds model that capture the odds of being at or above each VES score when continuous variables are centred at their median and discrete variables are set to their reference level (Note: for number of hospital and ER visits, values were centred at zero visits). ACS, acute coronary syndrome; ER, emergency room; MIDUS, Midlife Development in the United States; PHQ, Patient Health Questionnaire; s-TOFHLA, short form of the Test of Functional Health Literacy in Adults; VES, Vulnerable Elders Survey.

Figure 1 shows the results from the primary analyses using a multivariable proportional odds logistic regression model of the VES-13 score. Panel A shows the majority of variables for which there was little to no evidence of non-linearity; panel B shows depression, the only variable that exhibited non-linear effects; and panel C shows the estimated intercepts from the proportional odds model that capture the odds of being at or above each VES score when continuous variables are centred at their median and discrete variables are set to their reference level. The OR values represent the increased odds of a higher vulnerability (VES-13 score) for each patient variable as compared with the reference group. For example, a patient who has a diagnosis of ADHF has a threefold increased odds of higher vulnerability (OR 3.11, CI 2.06 to 4.70), compared with individuals with a diagnosis of ACS alone. Vulnerability was also independently and highly associated with being female (OR 1.55, CI 1.04 to 2.29) or widowed (OR 1.88 vs married, CI 1.06 to 3.34), and having being hospitalised in the prior 12 months (OR 1.30 per hospitalisation increase, CI 1.12 to 1.50). Further, a six-point increase in the ENRICHD social support score was associated with 1.42 OR (CI 1.10 to 1.84) of a higher VES-13 score. A lower VES-13 score (lower vulnerability) was associated with increased health literacy (OR 0.70, CI 0.56 to 0.88) and ER visits in the prior 12 months. In these adjusted analyses, there were no associations found with race, frailty indices or impaired cognition. Depression (figure 1B) demonstrated a very strong and non-linear relationship with vulnerability (p<0.001). In this figure, low PHQ-8 scores were associated with lower odds of vulnerability, whereas scores of 6 or more (mild, moderate and severe depression) were associated with significantly higher odds of vulnerability. Figure 1C shows the estimated intercepts from the proportional odds model that represent the odds of being at or above each VES-13 score when all other variables are fixed at the median value (continuous variables) and reference value (categorical variables). For hospital and ER visits, the reference values were centred at zero visits. For example, in our study population, an average white male (71 years of age, admitted with a diagnosis of ACS, not frail, not living alone, adequate health literacy, no cognitive impairment, no prior ER visits or hospital admission, and moderate social support) has 1.2 odds of having a VES-13 score of 2 or more.

Model results for secondary sensitivity analysis of VES-12 score demonstrated similar results. In this model, the relationships between independent predictors (depression, diagnosis of heart failure (HF) and prior hospitalisation) and the VES-12 outcome are similar to those shown in figure 1. Age was added as a covariate in this model and was not independently associated with VES-12 score. Predictors that were no longer significantly associated with the model (VES-12) included being female, widowed and reduced health literacy.

## Discussion

In this study, we demonstrated a high prevalence of vulnerability (54%) in older adults admitted with ACS and/or ADHF, which greatly exceeds the 32% prevalence reported in community-based cohorts.^22^ In addition, we found that a diagnosis of HF, recent hospitalisations, inadequate health literacy and depressive symptoms were highly associated with vulnerability and a short, self-report, function-based screening tool (VES-13) can be used to identify those individuals at particular risk for vulnerable functional status decline.

The association between HF and vulnerability to functional decline is consistent with prior reports.^36–38^ Underlying mechanisms of progression of HF share common origins with frailty in older adults,^39^ ^40^ and the chronic physically limiting nature of the HF syndrome may lead to reducing physical activity and worsening functional state. For older adults with HF, depression has been shown to be independently associated with future healthcare utilisation and mortality.^41^ Further, inadequate health literacy is associated with exacerbations of HF.^42^ The coalescing of these factors in association with vulnerable functional status suggests that hospitalised vulnerable adults with CVD, particularly HF, are not only at risk for a decline in functional status but are the most vulnerable to the worse outcomes.

We also demonstrated an association between vulnerability and significantly higher healthcare utilisation in the prior 12 months with the most vulnerable adults having a median of two hospitalisations suggesting that hospitalisation may be a significant driver of vulnerability. In the multivariable model, ER visits were no longer associated with vulnerability and an ER visit without hospital admission was associated with less vulnerability, suggesting the possibility that those individuals who presented to the ER but did not require hospital admission were less vulnerable. Further work from VICS will look to defining the relationship between the presence of vulnerability and future healthcare utilisation.

Implementing the VES-13 in an inpatient setting is a feasible tool to identify older adults who may stand to derive greater benefit from initiatives to prevent rehospitalisation, rehabilitation services or assessment for a major depressive illness that may be amenable to intervention. Objective measures are logistically difficult to implement in the inpatient setting where standards of CV care^19^ ^20^ (eg, bed rest, continuous cardiac monitoring, intravenous therapies and invasive procedures) may preclude their use, resulting in exclusion from testing of the most frail or at-risk patients.^18^ Indeed, although prior literature has demonstrated the highly predictive value of performance-based assessments in older hospitalised adults, the studies were limited due to the exclusion of patients with ACS and those who could not stand or complete the walk test.^16^

Another benefit of using the VES-13 in the hospital setting is its brevity, taking approximately 5 min to complete.^22^ It can be administered by nurses or non-clinical personnel, and could be incorporated as part of the nursing admission history to highlight deficits and trigger appropriate services, something which would not be as feasible for performance-based assessments. It can also provide a mechanism for clinicians and researchers to identify older adults who may particularly benefit from interventions aimed at preventing or slowing the progression of functional status decline following hospitalisations. An alternate approach to assessing patients’ morbidity involves use of comorbidity indices.^11^ ^43^ ^44^ We suggest that the self-perceived impact of declining health on function is more informative to a clinician than a list of comorbid conditions. Although beneficial in populations for predicting long-term outcomes,^11^ ^15^ the presence of a comorbid condition does not assess the physical and functional impact of the diagnoses. Understanding the functional limitations an individual perceives in his or her life provides more actionable information which can be addressed as part of transitional care planning.^45^

This study has limitations which include that it was performed at a single site that is inclusive of tertiary referral care for CVD which may restrict its generalisability. The parent study was designed to collect only self-reported information. This excluded some of the most ill patients, as well as those with hearing, vision or communication deficits; these groups might be expected to have a high prevalence of vulnerability. Thus, the true prevalence of vulnerability in the CVD population may actually be higher. The self-reported nature may also include some recollection bias in this elderly population, especially in relation to prior healthcare utilisation practices. The VES-13 does integrate self-perceived health, physical function limitations and IADL/ADL disabilities, but does not include an objective measure. The current study is a cross-sectional analysis and therefore, can only describe associations. For example, depression may not be a risk factor for vulnerability but, instead, vulnerability may be a risk factor for depressive symptoms.

To date, the VES-13 has primarily been utilised in only outpatient or community settings. In these studies, those individuals who were classified as vulnerable by score were 4.2 times more likely to decline or die in the 2 years following assessment and for each additional VES-13 point in a 5-year follow-up, the odds of the combined outcome of functional decline or death was 1.37.^21^ The VES-13 screening tool has not been utilised previously in acute care CV patients where the population is inherently more vulnerable during the acute episode, which may lead to possible overestimation of true baseline vulnerability prior to admission. It has, however, been shown to have predictive validity for significantly worse outcomes in older adults hospitalised with a traumatic injury,^23^ and for prioritising transition care in hospitalised older adults. We suggest that in older patients hospitalised with CVD, the VES-13 could be utilised to prioritise limited postacute care services aimed at preventing hospital readmissions. Future work from the VICS longitudinal data collection will include the association of baseline vulnerability with mortality, postdischarge healthcare utilization, and the mitigating effects of postacute care service utilisation.

## Conclusions

Vulnerability to functional status decline is highly prevalent in older adults admitted with ACS or ADHF, and is associated with other patient risk factors as well as an increased use of healthcare services. The VES-13 provides for an easily administered functional status screening tool that can be used to identify patients in the acute care setting who may require additional comprehensive assessments, further screening for diagnoses amenable to intervention, and prioritisation of postacute care services to address functional needs on discharge.
